# Engineered Cardiac Pacemaker Nodes Created by TBX18 Gene Transfer Overcome Source–Sink Mismatch

**DOI:** 10.1002/advs.201901099

**Published:** 2019-09-30

**Authors:** Sandra I. Grijalva, Jin‐mo Gu, Jun Li, Natasha Fernandez, Jinqi Fan, Jung Hoon Sung, Seung Yup Lee, Conner Herndon, Erin M. Buckley, Sung‐Jin Park, Flavio H. Fenton, Hee Cheol Cho

**Affiliations:** ^1^ Department of Biomedical Engineering Georgia Institute of Technology and Emory University Atlanta GA 30332 USA; ^2^ Department of Pediatrics Emory University Atlanta GA 30322 USA; ^3^ Department of Internal Medicine CHA Bundang Medical Center Seoul 13557 South Korea; ^4^ Department of Physics Georgia Institute of Technology Atlanta GA 30332 USA

**Keywords:** biological pacemakers, cell and tissue engineering, sinoatrial nodes, source–sink mismatch, TBX18

## Abstract

Every heartbeat originates from a tiny tissue in the heart called the sinoatrial node (SAN). The SAN harbors only ≈10 000 cardiac pacemaker cells, initiating an electrical impulse that captures the entire heart, consisting of billions of cardiomyocytes for each cardiac contraction. How these rare cardiac pacemaker cells (the electrical source) can overcome the electrically hyperpolarizing and quiescent myocardium (the electrical sink) is incompletely understood. Due to the scarcity of native pacemaker cells, this concept of source–sink mismatch cannot be tested directly with live cardiac tissue constructs. By exploiting TBX18 induced pacemaker cells by somatic gene transfer, 3D cardiac pacemaker spheroids can be tissue‐engineered. The TBX18 induced pacemakers (sphTBX18) pace autonomously and drive the contraction of neighboring myocardium in vitro. TBX18 spheroids demonstrate the need for reduced electrical coupling and physical separation from the neighboring ventricular myocytes, successfully recapitulating a key design principle of the native SAN. β‐Adrenergic stimulation as well as electrical uncoupling significantly increase sphTBX18s' ability to pace‐and‐drive the neighboring myocardium. This model represents the first platform to test design principles of the SAN for mechanistic understanding and to better engineer biological pacemakers for therapeutic translation.

## Graphical abstract

**The engineering sinoatrial node (eSAN) in a dish design** is inspired by the remarkable structure of the native SAN exit pathways. The architecture of the native SAN reduces the need for large number of pacemaker cells to spontaneously and autonomously pace‐and‐drive every single heartbeat.

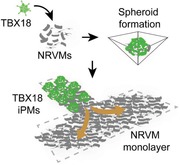

## 1 Introduction

More than a century ago, Keith and Flack discovered the sinoatrial node (SAN) as an anatomically distinct, minuscule tissue, in the heart at the boundary between the right atrium and the superior vena cava.^1^ Shortly after this anatomical discovery, the SAN was identified as the pacemaker of the heart, initiating each and every heartbeat.^2^ Electrically, the SAN and chamber myocardium are quite different. The atrial and ventricular cardiomyocytes are hyperpolarized at the reversal potential of K^+^ ions and resistant to depolarizing force owing to the strong inward rectifier ion channels such as Kir2.1 and Kir2.2.^3^, ^4^, ^5^ In contrast, the SAN nodal pacemaker cells lack *I*_K1_ and thus are relatively depolarized.^6^ Since there are less than ≈10 000 cardiac pacemaker cells in the SAN,^7^ how these few cardiac pacemaker cells can pace‐and‐drive the surrounding atrial myocardium has fascinated cardiac electrophysiologists for many decades.

For the SAN to initiate each heartbeat, it must overcome this mismatch between the electrical source and electrical sink.^8^ Earlier studies with sharp electrode recordings within the SAN have demonstrated that the cells within the core of the SAN are relatively uncoupled compared to the cells in the SAN periphery^9^, ^10^, ^11^ and in silico modeling has suggested that the weaker electrical coupling at the SAN core protect the initiation and facilitate the propagation of the automaticity.^8^, ^12^, ^13^, ^14^, ^15^ The SAN exhibits intricate architecture^7^, ^15^, ^16^, ^17^, ^18^, ^19^ which is thought to complement its function to pace‐and‐drive without fail. For example, the SAN appears to be electrically isolated from the atrial myocardium by connective and fibrotic tissue except the discrete exit pathways where SAN myocytes intertwine with atrial myocytes. 16, 20, 21, 22, 23, 24

Despite the principal importance of the SAN and the need to understand its function, tissue‐level models of the cardiac pacemaker have not been available. This is largely due to a very low yield of native pacemaker cells that can be isolated from the SAN, and the inability to culture them.^25^ As an alternative, pluripotent stem cells such as human and mouse embryonic stem cells have been employed to produce de novo cardiomyocytes with spontaneous and rhythmic contractions.^26^, ^27^, ^28^, ^29^ However, variabilities in cardiac differentiation conditions among different cell lines and the inherent heterogeneity of the derived cardiac myocytes are hurdles that impede progress.

We have previously demonstrated reprogramming of chamber cardiac myocytes to induced pacemaker cells by a transient expression of a single transcription factor, TBX18.^30^, ^31^, ^32^, ^33^, ^34^ The de novo TBX18‐induced pacemaker cells (iPMs) recapitulate hallmarks of native SAN pacemaker cells in their electrophysiology with their automaticity driven by the ion channels on the sarcolemma and the rhythmic releases of intracellular Ca^2+^.^31^ Here, we exploit TBX18 iPMs to generate 3D cardiac pacemaker spheroids in a defined shape and size.^35^ We patterned cardiac pacemaker tissue constructs into a defined structure in direct contact with monolayers of neonatal ventricular myocytes serving as electrical sinks. We tested the general hypothesis that TBX18 spheroids can pace‐and‐drive the electrical excitation of the neighboring ventricular myocytes as a model of source–sink mismatch in a dish. The results from this study form a foundation on which design principles of the native SAN can be reverse‐engineered for the eventual goal of creating tissue‐engineered SA nodes (eSANs).

## 2 Results

### 2.1 Spheroids of TBX18 iPMs Are Viable and Recapitulate Cardiac Pacemaker Gene Program

**Figure 1:**
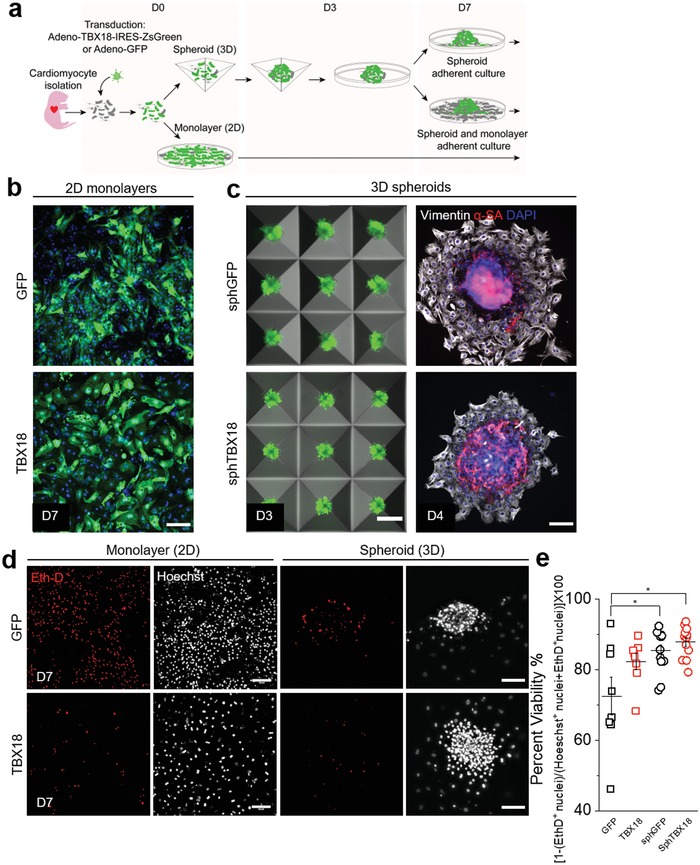
Characterization and generation of induced pacemaker spheroids. a) Timeline for generating spheroids from freshly isolated neonatal rat ventricular myocytes (NRVM) in Aggrewell plate. b) Representative green fluorescence reporter (GFP/ZsGReen) and Hoechst composite images of 2D culture. Scale bar 100 µm. c) First column: Generation of spheroids in AggreWell400 on day 3. Scale bar 200 µm. Second column: Representative immunostaining to denote location of α‐actinin (sarcomeric) cardiomyocytes and vimentin positive nonmyocytes once attached onto a glass substrate. Scale bar 100 µm. d) Representative maximum projection of epi‐fluorescence images of live dead staining using EthD‐1 in red and Hoechst in white of monolayer and spheroids on day 7. Scale bar 100 µm. e) Quantification yielding percent viability, *n* = 8–12 spheroids, *p < 0.05, one‐way ANOVA, mean ± SE.

Neonatal rat ventricular myocytes (NRVMs) represent the best characterized and validated platform to study cardiac physiology in vitro in a longitudinal manner.^36^ The NRVMs were freshly isolated from 1–3 d old pups and transduced with adenoviral vectors expressing either GFP (control) or TBX18 (experimental) to enable somatic gene transfer. The transduced NRVMs were seeded as either 2D monolayers or 3D cell aggregates by seeding them into an array of microwells (1000 cells per well) shaped in the form of inverted pyramids (**Figure** **1**a). Expression of the fluorescent reporter proteins, GFP in control NRVMs and ZsGreen in TBX18 induced pacemaker cells was robust in most myocytes, suggesting high efficiency of viral gene transfer (Figure 1b). The NRVM aggregates were suspended in culture media and allowed to form spheroids by gravity over 3 d (Figure 1c, D3), and then transferred to fibronectin coated vessels for longitudinal studies (Figure 1c, D4). Spheroids of cardiomyocytes may experience limited gas exchange at the core, which could negatively affect the cell viability and result in necrotic core. We examined cell viability by ethidium‐D homodimer (Eth‐D) staining on day 7, which binds to DNA of cells whose membrane have been compromised (Figure 1d). The viability of sphGFP and sphTBX18 were 85 ± 2 and 88 ± 1% (*n* = 12 each group), respectively, which were equal to or higher than the viability of GFP and TBX18 monolayers (72 ± 5 and 82 ± 2, respectively, *n* = 8 each group, *p < 0.05). Hence, the viability of spheroids was comparable to the viability of monolayers.

**Figure 2:**
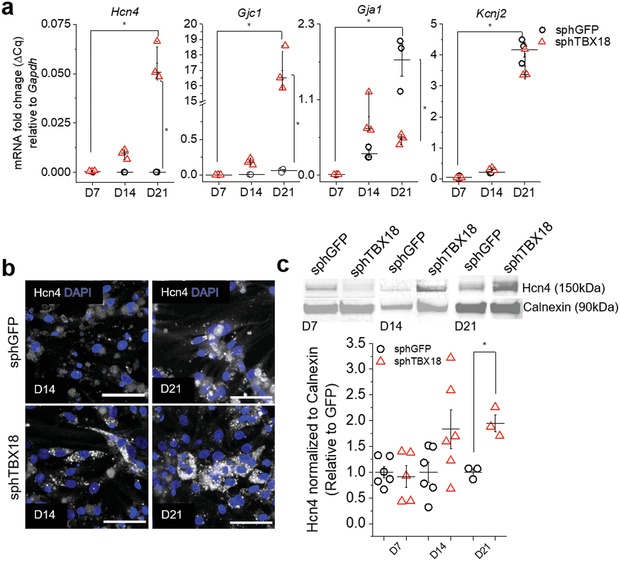
Characterization of pacemaker makers in induced pacemaker spheroids. a) Quantification of mRNA levels of selected genes normalized to *Gapdh* in spheres cultured in suspension using hanging drop over time (day 7, day 14 and day 21). Each reaction was performed from 10 spheroids, *n* = 3, *p < 0.05, one‐way ANOVA, mean ± SE. b) Representative immunostaining of Hcn4 in spheroids on day 14 and 21. Scale bar 50 µm. c) Representative western blot of spheroids on days 7, 14, and 21 (10 ug protein per lane) with Calnexin as the loading control. Quantification of Hcn4 protein (*n* = 3–6, *p < 0.05, one‐way ANOVA, mean ± SE).

Reprogramming of chamber cardiomyocytes to pacemaker cells by TBX18 leads to downregulation of ventricular and upregulation of nodal pacemaker gene programs.^31^, ^32^ We examined if the spheroid structure replicates this change. Expression of pacemaker marker genes such as *Hcn4* and *Gjc1* in sphTBX18 continually increased over 3 weeks while those in sphGFP remained minimally expressed. At D21 sphTBX18 spheroids kept in suspension showed 0.054‐fold higher *Hcn4* and 17‐fold higher *Gjc1* (which encodes Cx45) transcript levels compared to control (*n* = 3, *p* < 0.05). Conversely, expression of *Gja1*, which encodes connexin43 (Cx43), which is robustly expressed in chamber myocardium but not in the SAN, was 2‐fold lower in sphTBX18 compared to control. An inward rectifier potassium channel gene, *Kncj2*, was similarly expressed between sphTBX18 and sphGFP, suggesting that the K^+^ ionic current encoded by this gene would be comparable between the two groups (**Figure** **2**a). At the protein level, immunostaining revealed stronger expression Hcn4 ion channels in sphTBX18 compared to control on D14 and D21 (Figure 2b). Quantification by Western confirmed higher Hcn4 protein level in TBX18 spheroids compared to control at D21 (Figure 2c). Taken together, spheroids of TBX18 iPMs recapitulate the gene expression changes induced by TBX18 reprogramming.^31^

### 2.2 Electrical Coupling within TBX18 iPM Spheroids Resemble That of Native SAN

**Figure 3:**
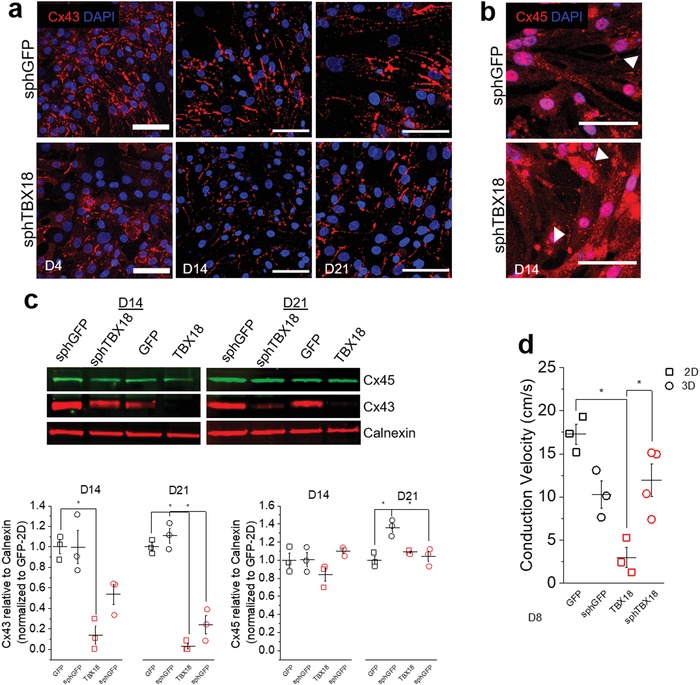
Gap junction protein expression and conduction velocity measurements. a) Representative immunostaining of Cx43 in spheroids on day 4, 14, and 21, scale bar 50 µm. b) Representative immunostaining of Cx45 in spheroids on day 14, scale bar 50 µm. c) Representative western blot of Cx43 and Cx45 for 2D and 3D groups on day 14 and 21 (10 ug protein per lane) with Calnexin as the loading control. Quantification of Cx43 and Cx45 protein, one‐way ANOVA from separate days, *p < 0.05 (mean ± SE, *n* = 3). d) Conduction velocity (cm s^−1^) from 2D or 3D cultures on day 8 (6‐well plate, 64 electrodes), one‐way ANOVA, *p < 0.05, *n* = 3–4 (*based on wells with synchronous beats) (mean ± SE).

The native SA node exhibits low cell‐cell electrical coupling at its core.^17^, ^37^ Reduced coupling is thought to help the pacemaker tissue overcome the surrounding, hyperpolarized atrial myocardium and facilitate propagation of the excitatory current.^8^, ^14^ Accordingly, high conductance gap junctions formed by Cx43 are found in the chamber myocardium, while the SAN's electrical coupling is mediated mainly by low conductance gap junctions formed by Cx45.^38^, ^39^ Expression of Cx43 proteins were robust and punctate in control sphGFP throughout the 3 weeks of continuous culture (**Figure** **3**a, upper panel). In contrast, intensity of Cx43 proteins were significantly weaker in sphTBX18 over the same time period (Figure 3a, lower panel). Cx45 protein expression did not appear to be different between sphGFP and sphTBX18 (Figure 3b). To complement the immunocytochemistry data with quantitative protein measurements, we performed Western on samples cultured for 7, 14, and 21 d. TBX18 spheroids and monolayers expressed significantly reduced levels of Cx43 proteins compared to sphGFP and GFP monolayers (Figure 3c, 14 ± 15% and 54 ± 17% reduced on D14, respectively, *n* = 3; 24 ± 15% and 3 ± 5% reduced on D21, respectively, *n* = 3, *p < 0.05). Protein levels of Cx45 were largely indistinguishable in TBX18 spheroids and monolayers compared to control spheroids and monolayers (Figure 3c). Western and immunostaining data were similar (Figure 3b) while our *Gjc1* (Cx45) transcripts levels were higher in sphTBX18 (Figure 2a, *Gjc1*). The inconsistency between transcript and protein levels of Cx45 may be biologically significant or could be related to known nonspecificity issues with Cx45 antibodies.

We asked if the significant reduction in Cx43 in sphTBX18 is reflected in slower conduction velocities (CVs). GFP or TBX18 spheroids were seeded on 64‐electrode multielectrode arrays (MEAs) at 300 spheroids per MEA (1000 NRVMs per spheroid). In line with the reduced expression levels of Cx43 by sphTBX18, the CV of TBX18 monolayers was significantly slower than that of control GFP monolayers (3 ± 2 vs 17 ± 2 cm s^−1^, *n* = 3, Figure 3d). However, the CV of sphTBX18 was not different from that of sphGFP (12 ± 6 vs 10 ± 3 cm s^−1^, respectively, *n* = 4 for sphTBX18 and *n* = 3 for sphGFP control, *p* < 0.05). This is likely due to the timing of our experiments; the CVs were measured in spheroids at D8, the time when Cx43 proteins were expressed at comparable levels between sphTBX18 and sphGFP (data not shown). Nonetheless, the data demonstrates that TBX18 spheroids show phenotypes of low cell‐cell electrical coupling, faithfully replicating the low electrical coupling within the native SAN.

### 2.3 TBX18 Spheroids Can Pace, and Its Synchrony Is Facilitated by Cocultured NRVMs

**Figure 4:**
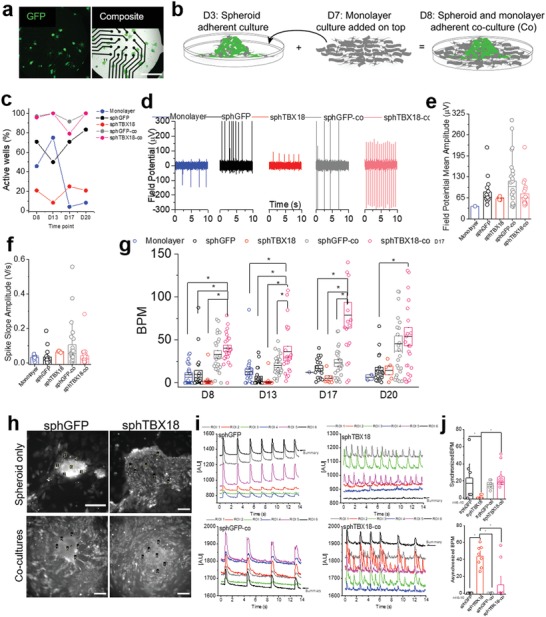
Characterization of electrophysiological synchronization of induced pacemaker spheroids. a) Green fluorescence reporter of single spheroids sparsely seeded on MEA and composite bright‐field images image on (day 4) (48‐well plate, 16 electrodes). Scale bar 1 mm. b) Representative schematic timeline of cocultures preparation. c) Percent active well (*n* = 24). d) Representative extracellular field potential (µV) recording on day 17. e) Quantification of extracellular field potential mean amplitude (µV) on day 17. f) Quantification of spike slope amplitude (V s^−1^) on day 17. g) Quantification of number of synchronized beating rates (BPM) on MEA over time (one way‐ANOVA on separate days, *p < 0.05, *n* = 24). h) Representative image of loaded intracellular Ca^2+^ dye (Rhod2AM) in spheroids and cocultures (spheroids and NRVM monolayer) and selected ROIs. Scale bar 100 µm. i) Spontaneously oscillating Ca^2+^ transients from multiple myocytes over time plotted simultaneously. j) Beats per minute (BPM) of synchronous beating (top) and asynchronous beating (bottom), *n* = 6–10, one‐way ANOVA, *p < 0.05, one‐way ANOVA (mean ± SE).

We examined spontaneous electrical activity of sphTBX18 on multiple 48‐well MEAs (16 electrodes per well). GFP or TBX18 spheroids were seeded sparsely on the MEA on D3 so as to detect electrical activities from no more than 1–2 spheroids per electrode (**Figure** **4**a). In another set of experiments, freshly isolated, nontransduced NRVMs were seeded over the spheroids on D7 in order to electrically couple all spheroids via NRVM monolayers and quantify their syncytial automaticity (Figure 4b). A total of 24 wells for each group were employed to monitor variability in synchronous beating. By D17 nontransduced NRVM monolayers became mostly quiescent while both sphGFP and sphTBX18 showed spontaneous activity with and without overlaid NRVM monolayers (Figure 4c). Examining the raw trace revealed that the amplitude of extracellular field potentials (FP) appeared to be lower for sphTBX18 compared to sphGFP (Figure 4d). Amplitude of FPs correlates to how tightly the myocytes are adhered to a given electrode and the number of electrically active myocytes close to it. Smaller FP amplitudes in sphTBX18 suggested that the iPMs may be loosely attached to the electrodes' surfaces and/or the individual iPMs' automaticity were not synchronized. Figure 4d,e illustrates that coculturing sphTBX18‐co with an overlay of nontransduced NRVMs slightly increased the FP amplitude compared to sphTBX18 alone. When cocultured with NRVM monolayers, the spike slope of FP was slower in sphTBX18 compared to sphGFP (Figure 4f), which is in line with the slow upstroke velocity of the action potentials from the SAN pacemaker cells compared to that from chamber cardiomyocytes. Examining the rate of spontaneous FPs indicate that sphTBX18 cocultured with NRVM monolayers showed the fastest rate over time compared to sphGFP, sphTBX18 and sphGFP‐co (Figure 4g, D13 and D17). Noting that sphGFP beating rate is susceptible to increase after media change (Figure 4g, D8 and D20). These data suggested that addition of NRVMs over TBX18 spheroids may have facilitated the individual iPMs to form a syncytium beating culture.

To better understand this phenomenon, we visualized oscillations of intracellular Ca^2+^ transients as a surrogate measure of individual myocytes' spontaneous electrical activity from sphGFP, sphTBX18, sphGFP‐co and sphTBX18‐co (Figure 4h; Videos S1–S4, Supporting Information). By randomly picking regions of interests, we plotted spontaneously oscillating Ca^2+^ transients simultaneously from multiple myocytes over time. The single myocytes from sphGFP and sphGFP‐co were well synchronized (Figure 4i left panels). In contrast, individual iPMs from sphTBX18 exhibited asynchronous electrical activities (Figure 4i top right), which was mitigated by addition of NRVM monolayers (sphTBX18‐co, Figure 4i bottom right). Indeed, addition of NRVM monolayers significantly increased the rate of synchronous automaticity and decreased the rate of asynchronous beats in sphTBX18 (Figure 4j). Taken together, TBX18 spheroids demonstrate pacemaker activity, and addition of NRVM monolayers facilitates their synchronous automaticity.

### 2.4 Generation of eSAN Model of Source–Sink Mismatch

**Figure 5:**
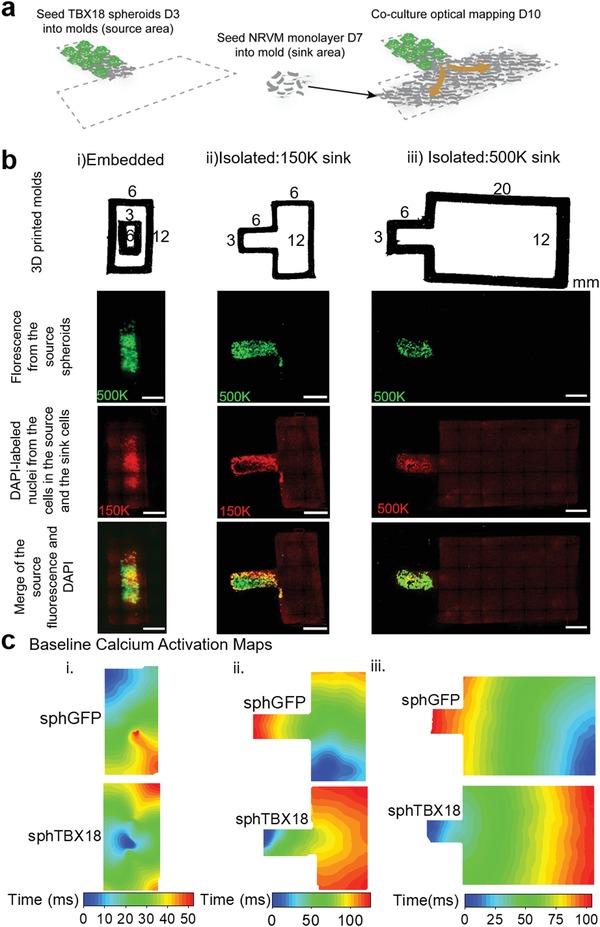
Generation of eSAN cocultures to monitor propagations of pace‐and‐drive with optical mapping. a) Schematic of cocultures achieved by patterning spheroids, depicted by green reporter, in desired location and naïve NRVM separately. b) Top row: 3D printed molds with dimensions for three designs. Second row: tile‐scan of the green fluorescence from the ZsGreen reporter in TBX18‐spheroids to indicate the source location. Third row: tile‐scan DAPI represents naïve untransduced NRVM nuclei and source nuclei (pseudocolor red). Fourth row: tile‐scan composite of green and DAPI. Scale bars 3 mm. c) Baseline calcium activation map of typical conduction propagations.

To test if TBX18 spheroids are able to pace‐and‐drive the neighboring myocardium, we patterned sphTBX18 within a rectangular shape to serve as an electrical ‘source' and juxtaposed monolayers of NRVMs as an electrical “sink” in fixed dimensions (**Figure** **5**a). While keeping the number of TBX18 or GFP spheroids constant in the source (1000 cells per spheroid, 150 spheroids per source), we created three different designs. Design one (i) focused on embedding “source” spheroids enclose by “sink” NRVMs (Figure 5b, i). In design two (ii), the sink NRVMs were seeded in contact with and next to the source spheroids (Figure 5b, ii). Design 3 was identical to design 2 except that the sink region was elongated (3X) (Figure 5b, iii). The spheroids in the source could be visualized by their respective reporter proteins (GFP for control and ZsGreen for TBX18, Figure 5b row 2), and 4′,6‐diamidino‐2‐phenylindole (DAPI) staining of the nuclei allowed visualization of all cells in the source and the sink (Figure 5b row 3, pseudocolored in red).

**Figure 6:**
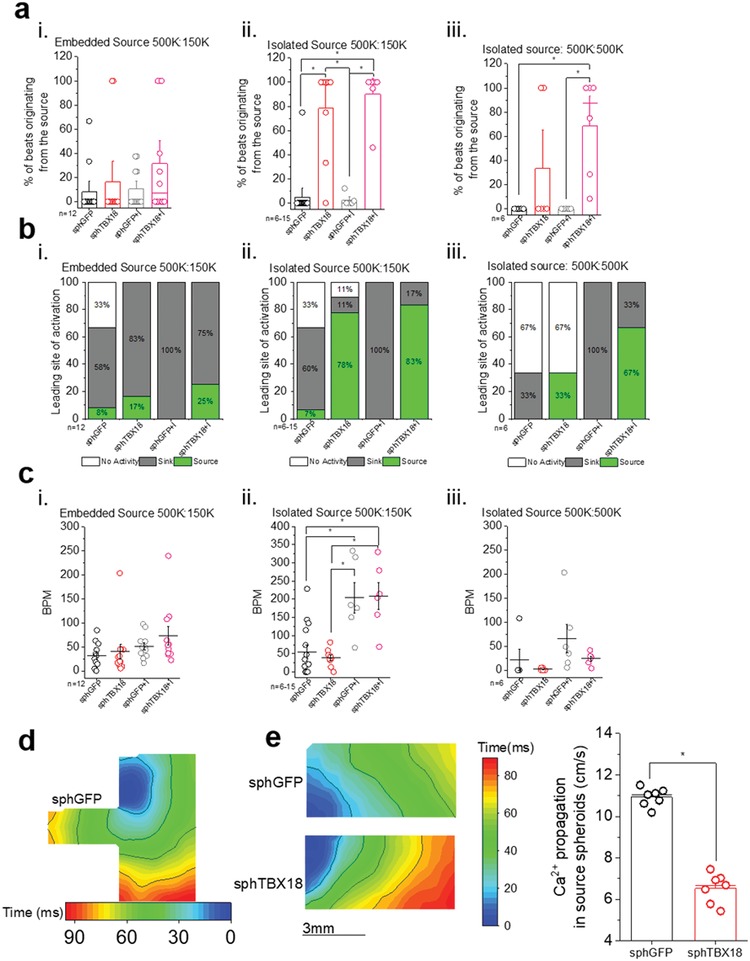
Quantification of eSAN coculture ability to pace‐and‐drive with optical mapping. a) Quantification of percent of beats originating from the source (spheres): Percent of beats originating from the source = 100% × (number of beats from source/total beats), one‐way ANOVA, *p < 0.05, mean ± SE. b) Classification of leading site of activation. If the percent of beats originating from the source was ≥50 than it is classified as “source,” <50 is “sink” and “no activity” was reserved for quiet cocultures. c) Quantification of beating rate (BPM), one‐way ANOVA, *p < 0.05, mean ± SE. d) Examples of re‐entrant arrhythmias such as self‐sustaining, fast rotors. e) Representative calcium activation map of source area (only spheroids) and quantification of Ca^2+^ propagation velocity (cm s^−1^) through source spheroids (Ca^2+^ propagation, cm s^−1^), *n* = 7, one‐way ANOVA, *p < 0.05, mean ± SE.

To identify the origin of electrical activation, we performed optical mapping of whole cell Ca^2+^ wave propagations by loading the entire tissue construct with an intracellular Ca^2+^ dye, Rhod2AM. (Figure 5c). In design (i), spontaneous Ca^2+^ waves mostly arose from the corners of the sink in sphGFP (Figure 5c, (i); Video S5, Supporting Information). A number of Ca^2+^ waves in sphGFP plus NRVM monolayer cocultures were indicative of reentrant arrhythmias such as self‐sustaining, fast rotors (**Figure** **6**d; Video S6, Supporting Information). In contrast, for sphTBX18 plus NRVM monolayer cocultures, Ca^2+^ waves were mostly originated from the sphTBX18 source and propagated out into the NRVM monolayer sink. Overall, the frequency of source‐to‐sink propagation was higher in design (ii) compared to design (i) (Figure 5c; Videos S7 and S8, Supporting Information). When the sink region was larger (design (iii)), the frequency of Ca^2+^ waves originating from the sphTBX18 source was lower compare to sphTBX18 cocultures in design (ii) (Figure 5c; Video S9, Supporting Information). This could be explained by the increased probability (increased area 3X) of automaticity to arise in the sink and overtake the source.

### 2.5 TBX18 Spheroids Can Pace‐and‐Drive, Serving as a Model of Source–Sink Mismatch

We analyzed the frequency of source‐to‐sink propagation in a quantitative manner. The mean percentage of beats originating from the spheroids (source) was calculated from three successive recordings of spontaneous Ca^2+^ propagations. In design (i), the percent of beats from the source was low for both groups: sphGFP (8 ± 6%, *n* = 12) and sphTBX18 (17 ± 11%, *n* = 12) (Figure 6a, (i), left two bars). In contrast, when the source has limited contact with the sink, but not embedded by the sink, (design (ii)), majority of the beats were initiated from spheroids in sphTBX18 cocultures (78 ± 13%, *n* = 9). However, the majority of the beats in sphGFP cocultures originated from the sink, NRVM cells (Figure 6a, (ii), left two bars). When the size of the monolayer sink was larger (design (iii)), the effectiveness of the source pacing decreased in sphTBX18 cocultures (33 ± 21%, *n* = 6) (Figure 6a, (iii), left two bars).

The native SAN's ability to respond to autonomic inputs is crucial for fight‐or‐flight response.^40^ We have previously demonstrated that TBX18‐iPMs exhibit improved beating rate response to β‐adrenergic stimulation.^31^ Indeed, treatment of the cocultures with a β‐adrenergic agonist, isoproterenol (ISO) at 1 × 10^−6^ m, increased the earliest site of activation originating from the source of sphTBX18 (32 ± 12%, *n* = 12) but none in sphGFP (10 ± 5%, *n* = 12) (Figure 6a, (i) right two bars). Similar trend was observed in designs (ii) and (iii) for sphTBX18 compared to sphGFP.

We classified the leading site of activation from source if the percent of beats originating from the source was ≥50%. While a percent less than 50% was quantified as sink, except if the cultures were nonresponsive and therefore classified as quiet (Figure 6b). When the source was embedded by the NRVM monolayers (Figure 6b (i)), most cocultures were driven by tachyarrhythmic activities from the NRVM monolayers (sink). When the source was separated from the NRVM monolayers with a single border, the majority of sphTBX18‐NRVM cocultures were paced from the TBX18 spheroids while control cocultures were largely the same as design (ii). Larger sink (design (iii)), decreased the number of cocultures with spheroid‐initiated pacing. Treatment with isoproterenol substantially improved the sphTBX18‐driven source‐pacing in design (iii), but had no effect on sphGFP‐NRVM cocultures (Figure 6b, (iii) right two panels). Our data indicate that β‐adrenergic stimulation by isoproterenol increased the frequency of electrical activities driven by TBX18‐spheroids in NRVM monolayer‐sphTBX18 cocultures. In contrast, the same treatment increased the frequency of spontaneous electrical activities in control, but the electrical activities were originated from the NRVM monolayers and not from GFP‐spheroids. Since β‐adrenergic signaling increases the production of cAMP which enhances gating of Hcn4 ion channels as well as intracellular Ca^2+^ cycling,^41^, ^42^ it is unclear what molecular determinants are responsible for the contrasting effects of isoproterenol treatment (Figure 6b, (i)–(iii)). Nonetheless, our data suggest that β‐adrenergic signaling enhances pace‐and‐drive function of sphTBX18, which suppresses ectopic electrical activities in the surrounding ventricular myocytes.

While a few cocultures were quiet, most of the ectopic activity we see in our sphGFP cultures includes rotors, which can be characterized as high BPM conductions (Figure 6c,i‐iii) in a circular manner (Video S6, Supporting Information). The beating rates of the cocultures were not significantly different among the groups (Figure 6c). This is likely due to the fact that control cocultures were spontaneously active due to tachyarrhythmias (Figure 6d, and Video S6, Supporting Information) rather than issues with sphTBX18 constructs. Specific response to isoproterenol supports this notion. We observed markedly slower Ca^2+^ propagation velocity within sphTBX18 compared to within sphGFP by optical mapping of the source area of design ii (Figure 6e, 6.54 ± 0.7 vs 10.9 ± 0.4 cm s^−1^, respectively, *n* = 7, *p* < 0.05). This is in line with our earlier study that TBX18 strongly suppresses chamber myocardial gap junction protein, Cx43,^32^ mimicking the slower conduction velocity in the native SAN.^22^

## 3 Conclusion

In this study, we engineered TBX18‐induced pacemaker spheroids and patterned the spheroids to model the SAN's ability to pace‐and‐drive the neighboring myocardium. The TBX18 spheroids paced at higher beating rates than control spheroids during prolonged coculture (Figure 4g). TBX18 spheroids could pace‐and‐drive a given sink when the spheroids were in contact with the sink in a single boundary, rather than being embedded by the monolayers (Figures 5 and 6). Notably, the TBX18 spheroids appear to exhibit different patterns of nonmyocyte organization (Figure 1b, right). The SAN consists of clusters of pacemaker cells surrounded by connective tissue strands such as collagen, elastin and fibroblast.^43^ Hence, the nonmyocyte organization within TBX18 spheroids warrant further investigation.

Earlier in silico simulation studies by Joyner and colleagues led to realization that the ability of the SAN, a tiny electrical source, to pace‐and‐drive the atrial myocardium is intricately dependent on the degree of nonlinear cell–cell coupling.^8^, ^12^, ^13^, ^14^ More recent simulations illuminate more design principles that facilitate the initiation and propagation of electrical activity by the SAN.^15^ These design principles include weak cell–cell electrical coupling in the core of the SAN,^44^ electrical insulation of the SAN by nonconducting tissue,^20^ regions of isotropic and anisotropic cell alignments,^17^, ^45^ and discrete exit pathways from the SAN out to the atrial myocardium.^21^ The SAN is located in the intercaval region of the right atrium, in between the crista terminalis and the septum. These both serve as physical electrical barriers due to their fibrous composition.^22^, ^43^, ^46^ The fat connective tissue also appears to isolate the SAN from the surrounding myocardium.^19^ For the first time, our data indicate that the TBX18 spheroid cocultures can serve as a platform to test the design elements of the native SAN. Our data successfully demonstrates that i) reducing the degree of electrical coupling between the source and the sink tissues facilitate pace‐and‐drive in sphTBX18 (Figure 3, 5, 6), and ii) specific and significant increase in the ability for iPM cells to pace‐and‐drive upon β‐adrenergic stimulation and iii) reduction physical connection to neighboring can help overcome the source–sink mismatch. Our 2–3 mm boundary length was derived from previous experimental data which showed pattered NRVM strands less than 55 µm in length cannot conduct and the known length of human exit pathways being around 3 mm.^16^, ^44^ One of the limitations of the current design is the spontaneous and often arrhythmic electrical activities from the NRVM monolayers. Overexpression of wild‐type Kir2.1 in NRVM monolayers and the ensuing increase in *I*_K1_ density should be able to minimize the activities in the control constructs.^3^, ^4^, ^5^, ^47^ In summary, TBX18‐induced pacemaker cell spheroids can overcome source–sink mismatch and serve as a platform to test the importance of the key features of the native SA node. Iterations of TBX18 pacemaker tissue constructs can then be studied in animal models of complete heart block for therapeutic development of biological cardiac pacemakers.^30^, ^48^, ^49^, ^50^ The insights gained will help understand the mechanisms of biological pacing in vivo, and facilitate the development of robust biological pacemakers.

## 4 Experimental Section

*Myocyte Isolation, Culture, and Adenoviral Transduction*: Neonatal rat ventricular myocytes (NRVMs) were isolated from 1 to 2 d old neonatal rat Sprague‐Dawley pups (Charles Rivers) as previously described.^31^ Three‐fourths of the ventricle was excised and treated with trypsin (Worthington Biochemical Corporation, Freehold, NJ) overnight and then enzymatically treated with collagenase (Worthington Biochemical Corporation, Freehold, NJ). Freshly isolated NRVMs were re‐suspended in M199 culture medium (Gibco, Waltham, MA) supplemented with 10% fetal bovine serum (FBS) (GE Healthcare, UK), 3.5 mg mL^−1^ glucose (Sigma, St. Louis, MO), 2 × 10^−3^ m GlutaMAX, 100 U mL^−1^ penicillin, 4 µg mL^−1^ vitamin B12, 10 × 10^−3^ m 4‐(2‐hydroxyethyl)‐1‐piperazineethanesulfonic acid (HEPES) buffer and 0.1 × 10^−3^ m minimum essential medium (MEM) nonessential amino acids (Gibco, Waltham, MA). Two 60 min preplating steps were performed to reduce fibroblasts and enrich cardiac myocyte content in the culture. NRVMs were transduced with Ad‐CMV‐GFP or Ad‐CMV‐TBX18‐IRES‐ZsGreen2 (MOI: 0.6–1.0) on the day of isolation for 2 h in suspension at room temperature and seeded on surfaces without removing the virus. Surfaces were previously coated with fibronectin 25 ug mL^−1^ (Corning, Corning NY). Monolayer cultures were seeded at a density of 21 000 cells cm^−2^. Cells were washed and media was changed 24 h after initial transduction. On day 2, media was replaced from 10% to 2% FBS and changed every other day.

*Spheroid Formation*: Spheroids were formed using Aggrewell 400 plates (STEMCELL, Vancouver, Canada). Aggrewell 400 plates consist of 24 wells with 1200 smaller microwells per well. 0.5 mL of Rinsing Solution (STEMCELL, Vancouver, Canada) was added to each well and the plate was centrifuged for 5 min at 2000 × *g*. Plate was washed twice with 1 mL phosphate‐buffered saline (PBS) prior to use and 0.5 mL of media was added to each well and centrifuged for 5 min at 2000 × *g*. NRVMs, previously transduced with virus, were suspended in media containing 10% FBS at a density of 1.0 × 10^6^ cells mL^−1^. 1.2 × 10^6^ NRVMs in a 1.2 mL volume was added to each well which resulted in 1000 cells per aggregated spheroid. The Aggrewell plate was centrifuged for 5 min at 10×*g*. Cell distribution across the well was verified under the microscope. The plate was incubated for three nights and media was exchanged every day by removing 0.5 mL from the well edge and adding 0.5 mL of fresh media. After three nights, the spheroids are formed and can be recovered from the plate by gently jetting them out with a pipette. The spheroids are separated from single cells by passing the spheroids through a 40 µm cell strainer (Corning, Corning NY) and reversing the filter to re‐suspend the spheroids in media.

*Cell Viability*: Fluorescence imaging was performed using Dmi8 Leica inverted microscope (Leica Microsystems, Wetzlar, Germany). Live dead staining was performed by incubating spheroids or monolayers cultured on glass plates with 10 × 10^−6^ m EthD‐1 (LIVE/DEAD Kit, Thermo, Waltham, MA) and 5 × 10^−6^ m Hoeschst (Thermo, Waltham, MA) in media, for 15 min at 37 °C. The dye was removed and new media was replenished. Oil fluorescence z‐stacks were acquired and maximum projection was constructed to input into CellProfiler (open source software) for analysis. Percent viability (PV) was defined as PV = 100 × [1 ‐ (EthD^+^ nuclei)/(Hoechst^+^ nuclei + EthD^+^ nuclei)].

*RT‐PCR*: Spheroid RNA was collected from spheroids kept in suspension using hanging drop method in order to maintain spheroid form from 1 to 3 weeks. Power SYBR Green Cells‐to‐Ct Kit was used to convert mRNA to cDNA from 10 spheroids (1000 cells per spheroid). The cDNA template was mixed with RT‐PCR cocktail and PCR was performed on the Rotor Gene Q (Qiagen, Hilden, Germany). The relative expression to Gapdh of the genes was calculated.

*Immunostaining*: Spheroid immunostaining was performed on spheroids seeded on glass or permanox plastic Lab‐Tek 8‐well chambers slides (Thermofisher, Waltham, MA) by washing culture twice with Dulbecco's phosphate‐buffered saline (DPBS) (Gibco, Waltham, MA) and incubating with 4% paraformaldehyde (Electron Microscopy Sciences, Hatfield, PA) for 15 min at room temperature.

Two immunostaining protocols were performed. The standard protocol consisted of blocking with 5% goat serum and permeabilizing with 0.3% Triton X‐100 for 1 h at room temperature. Samples were later incubated with primary antibody in 1% bovine serum albumin and 0.3% Triton X‐100 for 1 h at room temperature.

Anti‐Connexin45 sera detection required a different protocol, which consisted of 1 m glycine/PBS incubation for 30 min followed by three washes with PBS. A secondary fixing method was performed with 1:1 methanol/acetone for exactly 2 min at room temperature. Cells were washed three times with PBS and once with sodium borohydride 26 × 10^−3^ m NaBH4/PBS for 10 min. This was followed by permeabilization with 0.5% Triton X‐100 PBS for 10 min (two 5 min washes), and primary incubation in 2% goat serum for 1 h at room temperature.

Samples were stained with the following antibodies: anti‐α‐actinin (sarcomeric Sigma‐Aldrich; A7811; 1:400), anti‐HCN4 (Abcam; ab85023, 1:400), anti‐Connexin43 (Sigma‐Aldrich; C6219; 1:400), anti‐Vimentin (Abcam; ab24525, 1:600), and rabbit polyclonal antisera that recognizes Cx45 (1:800) was donated by Tom Steinberg's lab at Washington University School of Medicine in St. Louis.^51^ Alexa‐flour‐conjugated secondaries were added at ratio 1:400 (Thermo, Waltham, MA). All slides were mounted with Vectashield antifade mounting medium with DAPI (Vectro Laboratories, Burlingame, CA).

Slides were observed with laser scanning confocal imaging using Leica TCS SP8 (Wetzlar, Germany). The core of the spheroid was very dense with a height profile of >50 um, which made the core difficult to image using confocal, therefore boundary of spheroid was imaged.

*Western Blot*: One thousand spheroids were seeded on 24‐well plastic dishes on day 3 for prolonged culture in preparation for protein collection on days 7, 14, and 21. Spheroids were lysed on ice for 30 min with 100–125 µL of RIPA lysis and extraction buffer containing halt protease and phosphatase inhibitor mixture (ThermoFisher, Waltham, MA). Protein content was quantified using BCA assay and 10 µg samples were ran on 10% SDS‐polyacrylamide gels (Bolt, Thermo, Waltham, MA) and transferred onto FL‐PVDF membranes (Millipore sigma). The FL‐PDVF membranes were incubated with primary antibody overnight at 4 °C, followed by 1 h room temperature incubation with fluorophore‐conjugated secondary antibodies (680 and 800, Li‐cor corporate, Nebraska USA). Membranes were stained with the following antibodies: anti‐HCN4 (Alomone; APC‐052, 1:500), anti‐Connexin43 (Sigma‐Aldrich; C6219; 1:2000), and antisera for Connexin 45 as described in immunostaining section (1:500).^51^ Same membranes were stripped and re‐probed by monoclonal anti‐Calnexin antibody (Abcam, ab75801, 1:2000).

*Multielectrode Array Electrophysiology*: In vitro extracellular field potentials were recorded using MEAs at 37 °C and 5% CO2 condition using Axion BioSystems Maestro system with two plate types 48‐well (16 electrodes) or 6–12‐well (64 electrode) (Axion, Atlanta, GA). The raw signals were collected at 25 kHz, bandpass filtered (0.1 Hz to 2 kHz), and analyzed using Axion Cardiac Beat Detector using amplitude threshold of 40–100 µV to detect synchronized beats. Beating rates were calculated by taking the total number of synchronized beats and normalizing to the recording time (total beats dived by time = bpm). Wells were classified as active wells if they had more than 10 beats in over 10 min. The 64‐electrode array was used to calculate and create conduction maps with Axion Cardiac Conduction Tool.

*Calcium Recording*: Twenty spheroids were seeded on plastic ibidi 24‐well µ‐Plate with glass optical properties (ibidi, Germany) on day 3. On day 7, on subsequent NRVM isolation, NRVM monolayer was added to cocultured wells. On day 9, wells were incubated with 5 × 10^−6^ m Rhod2‐AM (ThermoFisher, Walthman, MA) with 0.02% Pluronic F127 for 30 min at 37 °C and then washed away and incubated at room temperature for 30 min. Calcium cycling videos were recorded on ORCA4 Flash at frame rate of 67 f s^−1^. Analysis was performed using ROI Manager on ImageJ.

*Optical Mapping*: Cocultures were achieved by seeding spheroids on 22 mm^2^ area plastic coverslips (VWR, Radnor, PA) on day 3 using various 3D printed custom silicon molds (Silicone 732, Dow, Midland, MI). Freshly isolated naïve NRVMs were added on day 7 and molds were removed on day 8. On day 9 or 10 cocultures were incubated in 5 × 10^−6^ m Rhod2‐AM (ThermoFisher, Walthman, MA) with 0.02% Pluronic F127 for 30 min at 37 °C. The dye was removed and the coverslips were incubated in Tyrode's solution for 30 min at room temperature. Tyrode's solution is composed of (in mmol L^−1^) 140 NaCl, 5.4 KCl, 1.2 KH2PO4, 5 HEPES, 1 MgCl2.6H_2_O, 5.5 Glucose, and 1.8 CaCl (pH 7.4).

A custom optical mapping system was built to record optical signals from large monolayer culture areas (3 × 3 cm^2^). Rhod2AM calcium dye (Thermofisher, Walthman, MA) was excited using a green LED (CBT‐90, Luminus Devices) with a 550 nm excitation filter (ET550/20x, Chroma). Fluorescence images were acquired at 500 f s^−1^ using Photometrics Evolve 128 EMCCD camera. A 596 nm emission filter (ET596+700LP, Chroma, Vermont, USA) was added between the camera and lens (Cosmicar TV Zoom Lens 8–48 mm 1:1.2). Photo toxicity damage was not observed across extended imaging.

Calcium propagation isochrones were constructed using a custom MATLAB code to average successive Ca^2+^ transients and determine the origin of activation and direction of the Ca^2+^ waves. Percent of Ca^2+^ waves, hereon referred as beats, originating from the source is defined by averaging a minimum of three recordings (14 s in length) for each sample as described in the following equation

(1) $$\text{Percent}\;\text{of}\;\text{beats}\;\text{originating}\;\text{from}\;\text{the}\;\text{source}\;=\;100\%\;\times \;\left[\frac{\text{Number}\;\text{of}\;\text{beats}\;\text{from}\;\text{source}}{\text{Total}\;\text{beats}}\right]$$

The leading site of activation was determined based from the value of percent of beats originating from the source. If the % was ≥50 than it is classified as “source”, < 50 is “sink” and “no activity” was reserved for quiescent cocultures. Isoproterenol hydrochloride (1 × 10^−6^ m, 51‐30‐9, Sigma) was used to stimulate the cocultures. Additionally, Ca^2+^ propagation velocity was quantified from the spontaneously beating source spheroids (design ii). Samples were chosen for data analysis when the Ca^2+^ propagations were originated from the source and spread to the sink. To control for beating rate‐dependent shortening of the action potential duration, samples with similar beating rates (≈150 bpm) were chosen. Seven independent propagations (*n* = 7) were quantified by calculating the slope of the activation map.

*Statistical Analysis*: Technical replicates were averaged to a single value. Values were expressed in mean and standard error (SE) unless otherwise specified. Data sets were statistically evaluated using student's two‐tailed paired t‐test, and one‐way ANOVA Tukey test, as appropriate. Confidence level of *p < 0.05 was considered significant unless indicated otherwise.

*Ethics Statement*: All procedures were approved by the institutional animal care and use committee (IACUC) at Emory University and executed in agreement with the guidelines for federal research published by the National Institutes of Health.

## Conflict of Interest

The authors declare no conflict of interest.

## Supporting information

Supplementary

Supplementary

Supplementary

Supplementary

Supplementary

Supplementary

Supplementary

Supplementary

Supplementary
